# Buffer or Brake? The Role of Sexuality-Specific Parenting in Adolescents’ Sexualized Media Consumption and Sexual Development

**DOI:** 10.1007/s10964-018-0828-3

**Published:** 2018-03-13

**Authors:** Geertjan Overbeek, Daphne van de Bongardt, Laura Baams

**Affiliations:** 1 Research Institute of Child Development and Education, University of Amsterdam, Amsterdam, The Netherlands; 2 Department of Psychology, Education and Child Studies, Erasmus University Rotterdam, Rotterdam, The Netherlands; 3 Pedagogy and Educational Sciences, Rijksuniversiteit Groningen, Groningen, The Netherlands

**Keywords:** Sexuality-specific parenting, Adolescence, Sexual development, Sexualized media consumption, Longitudinal

## Abstract

One main source of sexual socialization lies within family interactions. Especially sexuality-specific parenting may determine adolescents’ sexual development—adolescents’ sexual behavior and sexual risk behavior, sexualized media consumption and permissive sexual attitudes—to a significant extent, but different ideas exist about how this works. In this longitudinal study, we examined two hypotheses on how sexuality-specific parenting—parenting aimed specifically at children’s sexual attitudes and behaviors—relates to adolescents’ sexual development. A first *buffer hypothesis* states that parents’ instructive media discussions with their children—called instructive mediation—buffers the effect of sexualized media consumption on adolescents’ sexual attitudes and behavior and, vice versa, the effect of adolescents’ sexual attitudes and behavior on sexualized media consumption. A second *brake hypothesis* states that parents, by communicating love-and-respect oriented sexual norms, slow down adolescents’ development toward increased sexualized media use, permissive sexual attitudes, and sexual behavior and sexual risk behavior. Using four-wave longitudinal data from 514 Dutch adolescents aged 13–16 years (49.8% female), we found evidence to support a brake effect. More frequent parental communication of love-and-respect oriented sexual norms was associated with less permissive sexual attitudes and, for boys, with less advanced sexual behavior and a less rapid increase in sexual risk behavior. Parents’ instructive mediation regarding adolescents’ sexualized media consumption was associated with less permissive sexual attitudes at baseline, but only for girls. No systematic evidence emerged for a buffer effect of parents’ instructive mediation. In conclusion, although our data seem to suggest that parent–child communication about sex is oftentimes “after the fact”, we also find that more directive parental communication that conveys love-and-respect oriented sexual norms brake adolescents’ move toward sexual maturity.

## Introduction

Typically, as adolescents become older their sexualized media consumption increases (Savin-Williams and Diamond 2004), and they gradually develop more advanced sexual behavior and permissive attitudes (Crockett et al. 2003). These are normative trends that appear to be related. The more sexualized media adolescents consume, the more advanced they will be in their sexual development (Baams et al. 2015; Hennessy et al. 2009). One of the main sources of socialization, both of media consumption and sexual development, is parenting (De Graaf et al. 2011). But how does parenting determine the link between sexualized media consumption and sexual development in adolescence? This article examines two hypotheses related to this question. A first *buffer hypothesis* holds that parents buffer media effects on adolescents’ sexual development and, vice versa, buffer the effect of adolescents’ sexual attitudes and behavior on sexualized media consumption. A second *brake hypothesis* holds that parents, by communicating love-and-respect oriented sexual norms slow down adolescents’ development toward increased sexualized media consumption, permissive sexual attitudes, and sexual behavior and sexual risk behavior.

In this article, sexual behavior refers to sexual activities ranging from French kissing to intercourse, and sexual risk behavior refers to sexual activities that are potentially risky for adolescents’ health and mental health, such as having sex without a condom or performing sexual activities in front of a webcam. Although different manifestations of adolescents’ developing sexuality, sexual behavior and sexual risk behavior are related, because some sexually active adolescents may at some times engage in potentially risky activities (Kotchick et al. 2001), such as having unprotected sex or having sex with someone you just met.

### Sexualized Media Consumption and Sexual Development in Adolescence

Several theories explain how the development of sexualized media consumption and sexual behaviors and attitudes are intertwined. First, social cognitive theory (Bandura 1977, 1994) posits that we acquire knowledge about expected behaviors and scenarios by observing role models in social interactions. These effects are most prominent when a role model is similar and salient to the observer, and when a role model is rewarded for its behavior. Specific to sexualized media consumption, adolescents may acquire gender roles, knowledge about sexuality, a sexual behavior repertoire, and sexual attitudes based on sexualized media images (Hald et al. 2014). Indeed, some scholars argue that mass media function as a “sexual super peer” (Brown et al. 2005). When peer role models in mass media—in magazines, television shows, and on Internet—depict sex as appropriate for one’s age and as fun and worry-free, this encourages sexual behavior and sexual risk behavior and permissive sexual attitudes in adolescents. Adolescents may be especially susceptible to peer role models in popular media because they may lack a “reality check”, having relatively little sexual experience in real life (Brown et al. 2005).

Another relevant theoretical perspective, grounded in a transactional view of child–environment interactions, is that of selective exposure (Zillman and Bryant 1985). Selective exposure reasoning holds that one actively selects environments that support or confirm pre-existing perceptions and attitudes, and that one will tend to avoid environments that are incongruent with these perceptions and attitudes. Based on selective exposure reasoning, a media practice model has been put forward (Brown 2000). This model assumes that individuals’ existing attitudes or perceptions determine whether they will select or avoid specific media content. Thus, adolescents may actively select sexualized media based on their burgeoning sexual interests, or based on their first dating experiences and communications with peers about sexuality. The media practice model is circular, in that it specifies adolescents’ active role as media selectors, but also specifies that selection of specific media content will further strengthen and stimulate adolescents’ pre-existing sexual attitudes and behaviors.

Previous cross-sectional research has consistently identified associations between sexualized media consumption and more permissive sexual attitudes (e.g., Buhi and Goodson 2007; Brown and l’Engle 2009) and more advanced and risky sexual behaviors (e.g., Hald et al. 2013; Parkes et al. 2013). Only a few studies, however, have examined the bidirectionality of associations between sexualized media consumption and sexual development over time. One study, among Dutch 13–20 year olds, found that higher exposure to sexually explicit media content predicted more instrumental sexual attitudes (viewing sex as physical and casual, rather than as affectionate and relational) 6 months later (Peter and Valkenburg 2006). In addition, this study found that instrumental sexual attitudes predicted higher subsequent consumption of sexually explicit media content. Another study among Belgian 12–16 year olds found that more frequent visits to sexually explicit websites were related to earlier initiation of sexual intercourse, but here no reciprocal relation was found from sexual experience to the subsequent use of sexually explicit websites (Vandenbosch and Eggermont 2013). Finally, two studies that examined correlated change in sexualized media consumption and sexual development found that increases in sexualized media consumption go together with increases in permissive sexual attitudes (Baams et al. 2015) and sexual experience (Hennessy et al. 2009). Thus, previous research findings are in line with social cognitive and media practice models, showing positive and bidirectional relationships between sexualized media consumption and sexual development in adolescence.

### The Role of Sexuality-Specific Parenting: Buffer and Brake Effects

How does parents’ sexuality-specific parenting (parenting aimed specifically at children’s sexual attitudes and behaviors) determine links between adolescents’ sexualized media consumption and their sexual development? In this article we examine two possible hypotheses. A first *buffer hypothesis* holds that parents buffer sexualized media effects on adolescents’ sexual development, or vice versa buffer the selection and use of sexualized media content by adolescents who have previously become sexually interested and active. This buffering effect may be based on parental mediation: parents’ attempts to socialize, monitor, or restrict their child’s media use (Valkenburg et al. 1999). While parents’ use of restrictive mediation (i.e., controlling and limiting media use) may backfire—leading to more instead of less sexualized media consumption—in adolescents (Nathanson 2002), parents’ use of instructive mediation techniques (explaining and critically reflecting on media content) is generally considered effective in buffering effects of sexualized media consumption, because it may surpress the perceived realism of such media content in adolescents (Fisher et al. 2009). However, previous (mostly cross-sectional) studies have yielded inconsistent findings. For example, parents’ instructive mediation has been related to less stereotyped gender roles in adolescents who watched gender-stereotyped music videos (Nathanson et al. 2002), but other studies found no association between instructive mediation and adolescents’ sexual activity (Bersamin et al. 2008), or found that parent–child discussions about sexualized TV content predicted stronger—instead of weaker—intentions to have oral sex when adolescents were exposed to much sexualized content on TV (Fisher et al. 2009). Clearly, previous research has yielded inconsistent findings regarding a potential buffer effect of parents’ instructive mediation in the relationship between adolescents’ sexualized media consumption and their sexual development.

Another hypothesis that relates sexuality-specific parenting to adolescents’ sexual development is the *brake hypothesis*. The brake hypothesis holds that parents, specifically through communicating love-and-respect oriented sexual norms with adolescents, slow down the pace of development toward increased sexualized media use and permissive attitudes and sexual activity. Such a brake effect occurs when parents convey more love-and-respect oriented sexual norms to their offspring about having sex (for a review, see DiIorio et al. 2003; Dittus and Jaccard 2000). Parent–child communication about sex in general, however, is not unequivocally related to later onset or slower progression of sexual behavior (for a review, see Miller 2002). Previous findings on this issue are—again—contradictory. While some studies found more frequent parent–child communication about sex to predict less sexual experience of adolescents (Lehr et al. 2000), other studies found that parent–child communication about sex predicted more sexual experience (Jaccard et al. 1996; Ward and Wyatt 2006) and a higher likelihood of sexual initiation (Van de Bongardt et al. 2014). Yet other studies found no links between sexual experience and parent–child communication about sex (DiIorio et al. 2003; Jaccard et al. 2002).

### Current Study

In this four-wave longitudinal study among 13–16-year-old Dutch adolescents, we examined how adolescents’ sexualized media consumption and their sexual behavior and sexual risk behavior and permissive sexual attitudes were associated over time. We expected increases in adolescents’ sexualized media consumption to be associated with increases in three different indicators of adolescents’ sexual development: permissive sexual attitudes, sexual behaviors, and sexual risk behaviors. Second, we examined how parents’ instructive mediation *buffered* the links between adolescents’ sexualized media use and their permissive sexual attitudes and sexual behavior and sexual risk behavior. Third, we examined whether parent–child communication about sex, parental communication of love-and-respect oriented sexual norms, and parents’ instructive mediation *braked—*predicted less steep increases in—both sexualized media consumption and permissive sexual attitudes and sexual behavior and sexual risk behavior in adolescents. Previous research did not allow us to make specific hypotheses about gender differences in buffer or brake effects of sexuality-specific parenting; where possible we accounted for gender differences in the models.

The current study, to our knowledge, is the first longitudinal (2-year 4-wave) longitudinal examination of how sexuality-specific parenting is associated with adolescents’ sexual development over time. Importantly, this study bears information on a non-US context; one that is relatively liberal and characterized by an open, pro-active approach towards communicating about sex with adolescents (Weaver et al. 2005). Also, unlike many previous investigations, in this article sexual development is studied comprehensively: not focusing solely on adolescents’ sexual behavior and sexual risk behavior, but also focusing on adolescents’ sexual attitudes and sexualized media consumption.

## Methods

### Procedure and Sample

Ten secondary schools from various different regions in The Netherlands participated after checking all questionnaire content and learning about the 4-wave design of the study. All adolescents from selected classes—multiple classes per school participated—and their parents were then informed about the content and purpose of the study. Adolescents did not receive any compensation for their participation. Permission for the study was granted by the ethics board of the Faculty of Social Sciences of Utrecht University, under the study title “media use and development of adolescents”. Baseline (T_1_) questionnaires were administered in October–November 2009 by undergraduate students during regular school hours in the classroom, which took approximately 45 min Adolescents were told that their information would be handled confidentially and would not be shared with a third party (e.g., parents, teachers), and that they could withdraw participation at any time. For all items that referred to sex, adolescents could tick a box that said “I don’t want to answer.” At T_2_ (March–April 2010), T_3_ (October–November 2010), and T_4_ (March–April 2011) we used similar procedures. Schools’ participation was voluntary and schools did not receive compensation for their participation.

At baseline, 514 adolescents (50.2% boys) aged 13–16 years (M = 14.51; SD = 0.64) participated in the study. A total of 341 students (66.4%) followed pre-vocational education tracks, while 172 students (33.6%) followed senior general and pre-university education tracks. Further, 427 students (83.1%) had an indigenous Dutch background (adolescent and both parents born in the Netherlands), while 87 (16.9%) had various other ethnic backgrounds (adolescent or at least one parent not born in the Netherlands, e.g., Morocco, Turkey, Surinam, Indonesia, or Aruba). As for sexual orientation, 410 adolescents (81.7%) identified as heterosexual; 59 (11.2%) identified as gay/lesbian, 4 (0.8%) identified as bisexual, and 29 (5.8%) said they were unsure about their sexual orientation.

At T_4_, seven secondary schools still participated in the study (70%). The main reason for schools to stop participating was that most of the students who were included at T_1_ had graduated and left school. In most Dutch secondary schools, class composition—in terms of students following lessons together—changes quite drastically across the years. Thus, within schools we were unable to retain all T_1_ students in the longitudinal sample, as many were transferred to other classes. The high attrition rate, then, can be explained by school boards’ active replacement of students across classes in a school year rather than students’ active refusal to participate in the study. To acquire an optimal sample size, we only included classes at T_2_, T_3_, and T_4_ in which at least seven students had participated in the first wave. Of all baseline participants, 422 (82.1%) completed the questionnaire at T_2_, 271 (52.7%) at T_3_, and 220 (42.8%) at T_4_. A logistic regression analysis showed that adolescents following pre-vocational educational tracks were more likely to drop out of the study than adolescents following senior general and pre-university educational tracks (OR = 3.11, 95% CI = 2.09–4.62). None of the other sociodemographic background variables (religious background, age, ethnicity, and gender) predicted dropout from T_1_ to T_4_.

### Measures

#### Sexual development and sexualized media consumption (T_1_–T_4_)

##### Sexualized media consumption

Adolescents’ consumption of sexualized media images was measured with seven items, about how often in the past 6 months adolescents had looked into a sex or porn magazine, watched a porn video or DVD, watched a sex film on TV, watched an x-rated music video on the Internet, or looked at a porn website. Answering categories ranged from 1 (never) to 5 (very often). Scores on the items were averaged into one sexualized media consumption score per wave. A previous principal component analysis yielded a one-component solution for this scale, which demonstrated the construct validity of the instrument (Baams et al. 2015). Cronbach’s alphas ranged from 0.85 to 0.90 from T_1_ to T_4_.

##### Permissive sexual attitudes

Permissive sexual attitudes were assessed by measuring the extent to which adolescents agreed with ten statements of a sexually permissive nature (De Graaf et al. 2012). Adolescents responded on a 5-point scale, ranging from 1 (totally wrong) to 5 (totally right) to the following statement: “Imagine that you do the following things. What would you think of that?”. This was followed by items such as “you have sex with somebody you just met” and “you have sex for sex, not because you’re in love”. Thus, higher scores indicated more permissive sexual attitudes. Item scores were averaged into one permissive sexual attitude score. A previous principal component analysis provided evidence for the construct validity of this measure (Baams et al. 2015). The original scale includes 11 items, but we only used ten—excluding the item “you have sex before marriage” because in the current sociocultural context of The Netherlands a more positive score did not indicate that adolescents had more permissive sexual attitudes. Scores on the items were averaged into one permissive sexual attitudes score per wave. Cronbach’s alphas across measurements ranged from 0.86 to 0.90 from T_1_ to T_4_.

##### Sexual behaviors

The extent to which adolescents were sexually experienced was assessed with four items, pertaining to French kissing, touching and caressing, oral sex, and sexual intercourse. Answer categories ranged from 1 (never), to 2 (sometimes), and 3 (often). We first dichotomized scores on each item (0 = no experience, 1 = experience) and then summed all item scores into one sexual behavior score per wave. In doing so, we followed procedures employed in previous research (e.g., Doornwaard et al. 2015). Cronbach’s alphas ranged from 0.75 to 0.82 from T_1_ to T_4_.

##### Sexual risk behaviors

The extent to which adolescents engaged in risky sexual behaviors was assessed with eight questions, such as “stripped naked in front of a webcam”, “had sex without a condom”, and “had sex with someone you just met”. Answer categories ranged from 1 (never), to 2 (sometimes), and 3 (often). We again dichotomized scores on each item (0 = no experience, 1 = experience), and then summed the eight items into one risky sexual behavior score per wave. The Cronbach’s alphas ranged from 0.70 to 0.82 from T_1_ to T_4_.

#### Sexuality-specific parenting (T_1_)

##### Parent–adolescent communication about sex

At T_1_, adolescents reported on how often they perceived to talk with their parents about sex, based on 5 items for fathers and mothers separately (thus 10 items in total). The items pertained to themes of love and relationships, enjoyable sexual activities, sexual activities one is not ready for yet, pregnancy and contraceptives, and sexually transmitted infections (STIs). Answer categories ranged from 1 (never) to 5 (very often). Because correlations between father and mother items were high—*r*’s > 0.65, *p*’s < 0.001—father and mother item scores were averaged into one composite score. Cronbach’s alpha was 0.86.

##### Parents’ communication of love-and-respect oriented sexual norms

At T_1_, adolescents reported with four items how often their parents—this measure assessed perceptions for both father and mother—conveyed love-and-respect oriented sexual norms. The item stem specified: “Do your parents ever say that…”, example items were “… you should not have sex without being in love” and “… you should not engage in sexual activities that the other person does not want to engage in”. Scores of the items were averaged into one overall score. Answer categories ranged from 1 (never), 2 (sometimes) to 3 (often). Cronbach’s alpha was 0.72.

##### Parental instructive mediation

At T_1_, adolescents reported on how often they perceived their parents to discuss, or critically reflect on the media images they consumed and media functions they used. Specifically, they answered nine items, for instance: “My parents explain that you can’t always trust information from popular magazines” and “My parents talk with me about making dates through the Internet”, Item content of this scale conforms to that of similar scales used in previous research (e.g., Nathanson 2002). Answer categories ranged from 1 (never) to 5 (very often). Cronbach’s alpha was 0.73.

#### Statistical Analyses

Seven adolescents had missing data on all measurement waves and were therefore omitted from further analyses. Other missing values were handled using full information maximum likelihood procedures (Sass et al. 2014). To account for non-normality in our data we used the Robust Maximum Likelihood estimator, which corrects for deviation from multivariate normality by computing robust standard errors and an adjusted *χ*^2^ (Sass et al. 2014). In addition, we removed extreme outliers of +3 SD above the mean. Preliminary analyses (Fisher *r*-to-*z* transformation) did not show significant differences between lesbian, gay, bisexual, and transgender (LGBT) and non-LGBT adolescents in bivariate associations between sexuality-specific parenting and adolescents’ sexual development.

The main analyses were carried out in consecutive steps. In the first step, we performed descriptive analyses, examining sexual development and sexualized media consumption over time with latent growth curve analyses in Mplus (Muthén and Muthén 2010). We also examined gender differences in sexualized media consumption and sexual development indicators at each measurement wave using one-way ANOVA tests. In the second step, to examine how increases in adolescents’ sexualized media consumption were linked to increases in permissive sexual attitudes and (risky) sexual behavior, we estimated parallel process models in Mplus. Using these parallel process models, we obtained latent variables that represented intercepts (i.e., initial mean levels) and slopes (i.e., changes over time). Specifically, we assessed the variance in these intercepts and slopes, reflecting inter-individual variability in initial levels and changes over time (Duncan et al. 2013). We then examined correlated change over time between adolescents’ sexualized media consumption and sexual development by correlating their intercepts and slopes. Model fit was examined based on RMSEA and CFI indices.

In the third step, to examine whether parents’ instructive mediation buffered the link between adolescents’ sexualized media consumption and their permissive sexual attitudes and (risky) sexual behavior, we performed multi-group analyses. Specifically, we distinguished adolescents who scored relatively high (above the mean) and low (below the mean) on parents’ instructive mediation (*n* = 287 low; *n* = 168 high). To examine whether the buffering effect would be specific to parents’ instructive mediation or not, we also performed the multi-group analyses with parent–child communication about sex (*n* = 288 low; *n* = 170 high) and parents’ communication of love-and-respect oriented sexual norms (*n* = 271 low; *n* = 187 high). Satorra–Bentler chi-square difference tests were performed to compare unconstrained multi-group models, in which all parameters varied freely across high and low scoring groups, to models in which individual parameters were set equal between these groups. A significant chi-square increase indicated that parameters differed across groups, which would provide evidence for a buffer effect. Unfortunately, because of limited statistical power, no gender differences could be analyzed for buffer effects.

In the fourth step, to examine a “brake” effect of parent–child communication of love-and-respect oriented sexual norms, we entered this variable into parallel process models as predictor of the intercepts and slopes of adolescents’ sexualized media consumption and sexual development. To examine whether this brake effect would be specific to parents’ communication of love-and-respect oriented sexual norms, we also entered other sexuality-specific parenting behaviors as possible predictors: parent–child communication about sex and parents’ instructive mediation. We examined gender differences in these models by performing multi-group analyses for boys and girls. Chi-square difference tests were performed to compare unconstrained multi-group models to models in which individual parameters were set equal between genders. A significant increase in chi-square indicated a gender difference.

## Results

In order to examine the development of adolescents’ sexualized media consumption and permissive sexual attitudes and sexual behavior and sexual risk behavior from age 14–15 to 16–17, we estimated latent growth curve models for the total sample. All four models fit the data well, RMSEAs < 0.03, CFIs > 0.98. Overall, adolescents had relatively low levels of sexualized media consumption, which did not significantly increase over time (*M*_intercept_ = 1.42 (*p* < 0.001), *V*_intercept_ = 0.18 (*p* < 0.001), *M*_slope_ = −0.02 (*p* = 0.365, *V*_slope_ = 0.08, *p* = 0.012). No significant differences were apparent in sexualized media consumption between boys and girls (see Table 1). Significant variance (*V*) around the slope mean indicated that adolescents differed in how quickly they increased their sexualized media consumption. Adolescents developed increasingly permissive sexual attitudes over time (*M*_intercept_ = 1.96 (*p* < 0.001), *V*_intercept_ = 0.22 (*p* < 0.001), *M*_slope_ = 0.07 (*p* = 0.008), *V*_slope_ = 0.10 (*p* = 0.118), with boys endorsing significantly more permissive sexual attitudes than girls at each wave (see Table 1). The significant variance (*V*) around the intercept mean indicated that adolescents differed in their levels of permissive sexual attitudes at baseline. We observed significant increases in adolescents’ sexual behavior (*M*_intercept_ = 1.31 (*p* < 0.001), *V*_intercept_ = 1.47 (*p* < 0.001), *M*_slope_ = 0.33 (*p* < 0.001), *V*_slope_ = 0.54 (*p* < 0.001) and adolescents’ sexual risk behavior (*M*_intercept_ = 0.13 (*p* < 0.001), *V*_intercept_ = 0.18 (*p* < 0.001), *M*_slope_ = 0.07 (*p* = 0.003), *V*_slope_ = 0.17 (*p* = 0.001) over time. The significant variance around the slope means demonstrated that there were significant differences between adolescents in how quickly they increased their sexual behavior and sexual risk behavior. There were no significant gender differences apparent in the levels of sexual behaviors and sexual risk behaviors (see Table 1).

**Table 1:** One-way ANOVAs per measurement wave

	Boys		Girls			
	*M*	SD	*M*	SD	*F*	df_1_, df_2_
Sexualized media consumption						
T_1_	0.10	0.50	0.04	0.27	3.74	1, 502
T_2_	0.17	0.68	0.14	0.67	0.26	1, 498
T_3_	0.25	0.85	0.27	0.89	0.10	1, 468
T_4_	0.47	1.13	0.37	1.04	1.08	1, 435
Permissive sexual attitudes						
T_1_	2.16	0.64	1.81	0.45	46.41***	1, 453
T_2_	2.30	0.68	1.89	0.55	45.85***	1, 419
T_3_	2.34	0.65	1.85	0.51	58.97***	1, 332
T_4_	2.35	0.72	1.87	0.54	43.79***	1, 298
Sexual behavior						
T_1_	1.44	1.25	1.28	1.21	1.83	1, 439
T_2_	1.78	1.32	1.64	1.29	1.25	1, 408
T_3_	1.96	1.44	2.04	1.39	0.23	1, 317
T_4_	2.34	1.42	2.38	1.40	0.07	1, 282
Sexual risk behavior						
T_1_	0.26	0.91	0.18	0.49	1.41	1, 433
T_2_	0.41	1.19	0.26	0.64	2.35	1, 387
T_3_	0.56	1.29	0.41	0.82	1.40	1, 307
T_4_	0.86	1.63	0.68	1.32	1.05	1, 273

*** *p* < 0.001

### Parallel Process Models

The outcomes of the parallel process model analyses (see Table 2) were similar for boys and girls. The results showed that adolescents who consumed more sexualized media content at baseline were also more sexually experienced, reported more sexual risk behavior, and endorsed more permissive sexual attitudes at baseline. Importantly, adolescents who reported less sexual behaviors at baseline showed relatively steep increases in sexual behavior over time, compared with peers who at baseline were more advanced. This was not the case for sexual risk behaviors, however. Adolescents who at baseline reported more sexual risk behaviors showed relatively strong increases in sexual risk behaviors over time as well. Data also indicated that adolescents who at baseline reported more sexual behavior subsequently showed more limited increases in their sexualized media consumption. In a similar fashion, adolescents who at baseline reported more sexualized media consumption subsequently showed a less rapid progression into permissive sexual attitudes over time. In contrast, however, a higher level of sexualized media consumption at baseline was related to a steeper increase of sexual risk behavior in adolescents. Finally, regarding correlated change we found that adolescents who increased in sexualized media consumption also developed increasingly permissive sexual attitudes. No such correlated change was found between sexualized media consumption and sexual behavior and sexual risk behavior.

**Table 2:** Outcomes of parallel process models

	Permissive sexual attitudes		Sexual behavior		Sexual risk behavior	
	*B*	*p*	*B*	*p*	*B*	*p*
Boys						
Intercept SMC ←→ Intercept SDI	0.099^a^	<0.001	0.118	<0.001	0.031	<0.001
Slope SMC ←→ Slope SDI	0.002	0.041	0.003	0.198	<0.001	0.912
Intercept SMC ←→ Slope SMC	−0.030^a^	<0.001	−0.029^a^	<0.001	−0.030^a^	<0.001
Intercept SDI ←→ Slope SDI	−0.011	0.117	−0.080	0.007	0.032	<0.001
Intercept SMC ←→ Slope SDI	−0.007	0.026	0.003	0.667	0.018	0.001
Intercept SDI ←→ Slope SMC	−0.010^a^	0.054	−0.023	0.002	−0.003	0.407
Girls						
Intercept SMC ←→ Intercept SDI	0.044	<0.001	0.118	<0.001	0.031	<0.001
Slope SMC ←→ Slope SDI	0.002	0.041	0.003	0.198	<0.001	0.912
Intercept SMC ←→ Slope SMC	−0.012	<0.001	−0.012	<0.001	−0.012	<0.001
Intercept SDI ←→ Slope SDI	−0.011	0.117	−0.080	0.007	0.032	<0.001
Intercept SMC ←→ Slope SDI	−0.007	0.026	0.003	0.667	0.018	0.001
Intercept SDI ←→ Slope SMC	−0.002	0.582	−0.023	0.002	−0.003	0.407

←→ standardized correlation parameter, *SMC* sexualized media consumption, *SDI* sexual development indicator; fit for Permissive sexual attitudes model: *χ*^*2*^ (88, 455) = 180.939, *p* ≤ 0.001, RMSEA = 0.068, CFI = 0.906; fit for Sexual behavior model: *χ*^*2*^ (90, 455) = 271.582, *p* ≤ 0.001, RMSEA = 0.092, CFI = 0.885; fit for Sexual risk behavior model: *χ*^*2*^ (87, 455) = 174.728*, p* ≤ 0.001, RMSEA = 0.067, CFI = 0.917

^a^Significant gender difference: parameter set free to vary for boys and girls

### The Buffer Hypothesis

Overall, the multi-group analyses demonstrated that longitudinal associations and correlated changes between sexualized media consumption on the one hand, and permissive sexual attitudes and sexual behavior and sexual risk behavior on the other hand, were just as strong for adolescents who reported less or more instructive mediation from their parents (see Table 3). Across the different models tested, only a few (i.e., 7 out of 54 tests) multi-group tests yielded significant differences. Similar findings—no systematic pattern of multi-group differences—emerged for subgroups low versus high on parent–child communication about sex and for subgroups that were low versus high on parental communication of love-and-respect oriented sexual norms (Table 3). Because of limited statistical power, no gender differences were examined.

**Table 3:** Outcomes of buffer models: parallel Process models for below and above-average groups on parenting predictors

	Permissive sexual attitudes		Sexual behavior		Sexual risk behavior	
	*B*	*p*	*B*	*p*	*B*	*p*
*Below-mean groups*						
Parent–adolescent sex communication						
Intercept SMC ←→ Intercept SDI	0.617	<0.001	0.349	<0.001	0.173	0.003
Slope SMC ←→ Slope SDI	0.338	0.009	0.065	0.411	0.004	0.968
Intercept SMC ←→ Slope SMC	−0.551	<0.001	−0.563	<0.001	−0.559	<0.001
Intercept SDI ←→ Slope SDI	−0.124	0.312	−0.169	0.003	0.168^a^	0.059
Intercept SMC ←→ Slope SDI	−0.256^a^	0.013	0.017	0.776	0.184	0.008
Intercept SDI ←→ Slope SMC	−0.115	0.271	−0.214	0.006	−0.106	0.250
Parental communication of LAR sexual norms						
Intercept SMC ←→ Intercept SDI	0.538	<0.001	0.337	<0.001	0.156	0.004
Slope SMC ←→ Slope SDI	0.272	0.036	0.099	0.168	−0.019	0.781
Intercept SMC ←→ Slope SMC	−0.487	<0.001	−0.496	<0.001	−0.490	<0.001
Intercept SDI ←→ Slope SDI	−0.102	0.427	−0.195	0.002	0.252	0.001
Intercept SMC ←→ Slope SDI	−0.072	0.402	−0.085^a^	0.182	0.160	0.003
Intercept SDI ←→ Slope SMC	−0.079	0.397	−0.211	0.003	−0.099	0.223
Parental instructive mediation						
Intercept SMC ←→ Intercept SDI	0.537^a^	<0.001	0.365	<0.001	0.079^a^	0.186
Slope SMC ←→ Slope SDI	0.315	0.014	−0.030^a^	0.743	0.002	0.979
Intercept SMC ←→ Slope SMC	−0.558	<0.001	−0.562	<0.001	−0.557	<0.001
Intercept SDI ←→ Slope SDI	−0.088	0.498	−0.177	0.003	0.166^a^	0.055
Intercept SMC ←→ Slope SDI	−0.116	0.199	0.019	0.740	0.207	0.001
Intercept SDI ←→ Slope SMC	−0.147	0.114	−0.245	<0.001	−0.087	0.277
*Above-mean groups*						
Parent–adolescent sex communication						
Intercept SMC ←→ Intercept SDI	0.479	<0.001	0.270	<0.001	0.134	0.003
Slope SMC ←→ Slope SDI	0.254	0.006	0.063	0.411	0.003	0.968
Intercept SMC ←→ Slope SMC	−0.322	<0.001	−0.343	<0.001	−0.342	<0.001
Intercept SDI ←→ Slope SDI	−0.124	0.312	−0.208	0.003	0.538^a^	<0.001
Intercept SMC ←→ Slope SDI	0.086^a^	0.418	0.016	0.777	0.142	0.009
Intercept SDI ←→ Slope SMC	−0.086	0.282	−0.169	0.011	−0.084	0.247
Parental communication of LAR sexual norms						
Intercept SMC ←→ Intercept SDI	0.538	<0.001	0.337	<0.001	0.156	0.004
Slope SMC ←→ Slope SDI	0.272	0.036	0.099	0.168	−0.032	0.781
Intercept SMC ←→ Slope SMC	−0.487	<0.001	−0.469	<0.001	−0.490	<0.001
Intercept SDI ←→ Slope SDI	−0.102	0.427	−0.195	0.002	0.420	<0.001
Intercept SMC ←→ Slope SDI	−0.072	0.402	0.149^a^	0.108	0.267	0.002
Intercept SDI ←→ Slope SMC	−0.079	0.397	−0.211	0.003	−0.099	0.223
Parental instructive mediation						
Intercept SMC ←→ Intercept SDI	0.594^a^	<0.001	0.303	<0.001	0.240^a^	<0.001
Slope SMC ←→Slope SDI	0.315	0.014	0.236^a^	0.025	0.002	0.979
Intercept SMC ←→ Slope SMC	−0.452	<0.001	−0.465	<0.001	−0.450	<0.001
Intercept SDI ←→ Slope SDI	−0.088	0.498	−0.208	0.003	0.653^a^	<0.001
Intercept SMC ←→ Slope SDI	−0.094	0.201	0.019	0.741	0.167	0.002
Intercept SDI ←→ Slope SMC	−0.147	0.144	−0.245	<0.001	−0.087	0.277

*Parental communication of LAR sexual norms* Parental communication of love-and-respect oriented sexual norms, ←→ standardized correlation parameter, *SMC* sexualized media consumption, *SDI* sexual development indicator

^a^Significant group difference: parameter set free across groups of males and females; fit for the Permissive sexual attitudes models: Parent–adolescent sex communication: *χ*^*2*^ (53, 458) = 102.872, *p* ≤ 0.001, RMSEA = 0.064, CFI = 0.944; Parental communication of sexual norms: *χ*^*2*^ (56, 458*)* = 141.074, *p* ≤ 0.001, RMSEA = 0.081, CFI = 0.908; Parental instructive mediation; *χ*^*2*^ (54, 455) = 108.117*, p* ≤ 0.001, RMSEA = 0.066, CFI = 0.936; fit for the Sexual behaviors models: Parent–adolescent sex communication: *χ*^*2*^ (55, 458) = 137.554, *p* ≤ 0.001, RMSEA = 0.081, CFI = 0.939; Parental communication of sexual norms: *χ*^*2*^ (57, 458) = 163.284, *p* ≤ 0.001, RMSEA = 0.090, CFI = 0.924; Parental instructive mediation; *χ*^*2*^ (55, 455) = 149.094, *p* ≤ 0.001, RMSEA = 0.087, CFI *=* 0.931; fit for the Sexual risk behaviors models: Parent–adolescent sex communication: *χ*^*2*^ (55, 457) = 74.256, *p* = 0.043, RMSEA = 0.039, CFI = 0.976; Parental communication of sexual norms: *χ*^*2*^ (57, 457) = 100.777, *p* ≤ 0.001, RMSEA= 0.058, CFI = 0.949; Parental instructive mediation; *χ*^*2*^ (55, 454) = 95.826, *p* ≤ 0.001, RMSEA = 0.057, CFI = 0.947

### The Brake Hypothesis

For both boys and girls, sexuality-specific parenting at T_1_ was associated with adolescents’ sexual development from T_1_ to T_4_ (see Table 4). Specifically, more frequent parent–child communication about sex was associated with more sexualized media consumption and more adolescent-reported sexual activity at baseline and to increases in sexual risk behavior over time. However, this latter finding emerged for boys only. Also for boys but not for girls, we found that more frequent parental communication of love-and-respect oriented sexual norms were associated with less permissive sexual attitudes at baseline, and to a slower progression of sexual risk behavior in adolescents over time. Overall, then, evidence for a brake effect of sexuality-specific parenting was found in boys. Further analyses demonstrated, also, that results were specific to parent–child communication of love-and-respect oriented sexual norms. That is, parent–adolescent communication about sex was associated with higher, not lower, levels of permissive sexual attitudes and (increases in) sexual activity in adolescents, and parents’ instructive mediation was not associated with baseline levels and development of adolescents’ permissive sexual attitudes and sexual behavior and sexual risk behavior—except for one finding, demonstrating that girls reported less permissive sexual attitudes at baseline when they had received more instructive mediation from their parents.

**Table 4:** Outcomes of brake models: parallel process models with parameter estimates for sexuality-specific parenting variables

	Parent–adolescent sex communication		Parental communication LAR sexual norms		Parental instructive mediation	
	*B*	*p*	*B*	*p*	*B*	*p*
*Boys*						
Permissive sexual attitudes						
Intercept	0.088	0.054	−0.191^a^	<0.001	−0.013^a^	0.306
Sexual behavior						
Intercept	0.555	<0.001	0.064	0.537	−0.149	0.263
Slope	−0.046	0.278	0.037	0.334	−0.052	0.193
Sexual risk behavior						
Intercept	0.028	0.599	−0.075^a^	0.051	0.015	0.745
Slope	0.165^a^	0.021	−0.087^a^	0.048	−0.059	0.062
*Girls*						
Permissive sexual attitudes						
Intercept	0.088	0.054	−0.053	0.280	−0.078	0.041
Sexual behavior						
Intercept	0.555	<0.001	0.064	0.537	−0.149	0.263
Slope	−0.046	0.278	0.037	0.334	−0.052	0.193
Sexual risk behavior						
Intercept	0.028	0.599	0.090	0.089	0.015	0.745
Slope	0.056	0.075	−0.016	0.569	−0.059	0.062

No parameter estimates are presented for the Permissive sexual attitudes slope, because no significant variance was found around its mean slope; Parental communication of LAR sexual norms = Parental communication of love-and-respect oriented sexual norms; Parameter estimates *B* and *p* for Sexualized media consumption differed across three parallel process models (for different indicators of adolescent sexual development: Permissive sexual attitudes, Sexual behavior, and Sexual risk behavior, respectively)—in all models, a higher baseline level (intercept) of Sexualized media consumption was significantly predicted by more frequent parent–adolescent communication about sex (*B*s > 0.15, *p*s < 0.003), not by parental communication of love-and-respect oriented sexual norms or parental instructive mediation. A less steep increase (slope) in Sexualized media consumption was significantly predicted by more frequent parent–adolescent communication about sex (*B* = −0.031, *p* < 0.001) in the Sexual behavior model, and by more frequent parental communication of love-and-respect oriented sexual norms in the Permissive attitudes model (*B* = −0.179, *p* < 0.001)

^a^Significant gender difference: parameter set free for boys and girls

## Discussion

Typically, as adolescents become older their sexualized media consumption increases (Savin-Williams and Diamond 2004), and they gradually develop more advanced sexual behavior and permissive attitudes (Crockett et al. 2003). Sexual development in adolescence is a socialized phenomenon, and for an important part adolescents’ sexual socialization is shaped in family interactions. Thus, sexuality-specific parenting may determine adolescents’ sexual development—specifically adolescents’ sexual behavior and sexual risk behavior, sexualized media consumption and permissive sexual attitudes—to a significant extent. But how does this work exactly?

The present study examined two hypotheses on the role of sexuality-specific parenting in adolescents’ sexualized media consumption and sexual development. A first “buffer hypothesis” stated that specifically parents’ instructive media discussions with their children—called instructive mediation—buffers the effect of sexualized media consumption on adolescents’ sexual attitudes and behavior, and vice versa. A second “brake hypothesis” stated that parents, through communicating love-and-respect oriented sexual norms, slow down adolescents’ development toward increased sexualized media use and sexual behavior and sexual risk behavior. Using 18-month, four-wave longitudinal data from 514 adolescents aged 14–16 years, we found evidence to support a brake effect. More frequent parental communication of love-and-respect oriented sexual norms was associated with less permissive sexual attitudes, and for boys was associated with a less steep increase in sexual risk behavior. In contrast, however, parent–child communication about sex was related to more sexual behavior in boys and girls, as well as to a stronger increase in sexual risk behavior in boys. No evidence emerged to support a buffer effect of parents’ instructive mediation.

Results from the parallel process models showed that adolescents who reported less advanced sexual behavior at baseline demonstrated a relatively steep subsequent increase in sexual behavior across the four measurement waves. This suggests that over time, “late bloomers” may catch up with their earlier starting peers over the course of middle adolescence. This catch-up effect was evident in another way as well: adolescents who at baseline demonstrated lower levels of sexualized media consumption than their peers showed relatively steep increases in sexual behavior over time. This is readily explained by the positive association between sexualized media consumption and sexual development indicators in adolescents (Peter and Valkenburg 2006; Vandenbosch and Eggermont 2013): an advanced developmental position on one indicator signals an advanced position on another as well. These findings link back to social cognitive and media practice models about the relationship between adolescents’ sexualized media consumption and sexual attitudes and behaviors, and demonstrate that adolescents’ sexualized media consumption on the one hand and permissive sexual attitudes and sexual behavior and sexual risk behavior on the other hand, develop in tandem (see also Baams et al. 2015; Hennessy et al. 2009)—like different components of one underlying move towards sexual maturity. Importantly, although adolescents’ risky sexual behavior cannot be considered normative, our findings do show that sexual risk behavior is more likely to occur with an increasing level of sexual activity over the course of adolescence.

In line with previous study findings, we found a positive association between parent–child communication about sex and adolescents’ sexual behavior (Jaccard et al. 1996; Van de Bongardt et al. 2014; Ward and Wyatt 2006), as well as adolescents’ sexual risk behavior. One logical explanation for this finding may be that it demonstrates a child effect. Adolescents’ first steps in the developmental domains of love and sexuality will, in many families, not go unnoticed. Asking someone out for a date, falling in love, or exploring one’s sexual identity may be openly disclosed by adolescents or picked up by parents, evoking parent–child conversations or discussions about various sexuality-related topics (Jaccard et al. 2002). The present analysis also makes clear, however, that when parents do not “just talk” about sex with their children, but specifically convey more love-and-respect oriented sexual norms (such as not not having casual sex, or not having sex with someone you just met) this puts a brake on the developmental pace toward higher levels of sexual activity. This effect was particularly strong for boys. Why this is so remains a topic of further inquiry, but perhaps an explanation is that because men have a specifically strong biological sex drive (Baumeister et al. 2001)—associated with a relatively strong drive to engage in sex and casual sexual behaviors—parenting practices that limit the expression of this drive are especially effective for them. Another explanation may be found in the sexual double standards for boys and girls. Because parents’ evaluation of sexual activity may be less positive for adolescent girls than boys (Kreager and Staff 2009; Lyons et al. 2011), leading to more restrictive parenting for girls in the first place, sexuality-specific parenting that emphasizes love, restraint, and health may be more salient for boys and thus affect them more.

Our results did not support moderating effects of parents’ instructive mediation in the longitudinal associations between adolescents’ media consumption and their sexual development. At first sight, this finding seems to contrast the outcomes of previous research (e.g., Fisher et al. 2009; Nathanson et al. 2002), and the general notion that instructive mediation may be an effective parenting strategy to modulate effects of popular media on adolescent development (Valkenburg et al. 1999). However, our present findings do not necessarily imply that parents’ use of instructive mediation is not a viable strategy for preventing the development of risky sexual attitudes and behaviors in adolescents. It may well be that the effects of instructive mediation and parental media critique are relevant, but mostly when delivered in warm, supportive family contexts. Because our present analyses did not incorporate the broader parenting context, we cannot provide any definitive conclusion about this matter. It follows that future investigations should focus not only on the frequency of conversations about sexuality and media use between parents and their adolescents, but also on aspects such as timing, responsiveness, and comfort level of parents in sexuality-specific parenting, and the general affective quality of the parent–adolescent relationship.

Related to the effects of parents’ instructive mediation, also, the moderator analyses in our present study yielded a suprising finding. Specifically, increases in adolescents’ sexualized media consumption were more strongly linked to increases in sexual behavior for adolescents who received more—instead of less—instructive mediation from their parents. Perhaps, parents’ instructive mediation may inadvertently signal to adolescents that although they should be critical of what they see, they are not prohibited to consume sexualized media content. Especially with increasing levels of sexualized media consumption, which is predictive of an increased level of perceived realism of such images (Peter and Valkenburg 2010), it may be that mere exposure to sexualized media may still stimulate adolescents’ move towards increasing sexual activity, thwarting the effect of critical reflection and discussion of sexualized media content by parents. This may be especially true given that the frequency with which parents (according to adolescents) engaged in instructive mediation in our present study was relatively low—which corroborates findings from previous research showing that parent–adolescent communication about sex occurs relatively infrequently (Van de Bongardt et al. 2014).

Some limitations of this study warrant mentioning. First, the analyses were conducted using data from on a sample mostly consisting of adolescents with a Dutch background. Because previous research suggested that the impact of sexualized media images may differ across ethnic groups (Hennessy et al. 2009), it is unclear to what extent these results can be generalized across cultures or adolescents’ gender. Regarding this last point, it is essential that future studies examine in more detail how sexuality-specific parenting of fathers and mothers affects boys and girls differently. Indeed, gender intensification theory (Hill and Lynch 1983) and emprical research suggests that supportive attitudes towards romantic and sexual experimentation may be sex-linked, and more pronounced in father–son and mother–daughter dyads (Shulman et al. 2017). Second, our present study was not informative specifically about the development of LGBT adolescents, but rather dealth with adolescents with mostly heterosexual identities. A third limitation is that our study relied on adolescent self-reports. Although this is necessary given the highly personal and sensitive nature of the topic—reflected in our study by parent–child communication about sex hovering between “never” and “sometimes”—it does allow for alternative explanations based on uni-informant bias. With regard to sample attrition, the attrition from T_1_ to T_4_ was sizeable—resulting in a relatively small *n* (220) at the last measurement wave. Finally, based on our findings, we were unable to identify whether specific sexual behaviors occurred in the context of a romantic relationship or not. Having this information would have improved a differentiation between sexual behaviors and sexual risk behaviors, which in our current analyses are to a certain extent conflated. Despite these limitations, we feel this study yielded valuable results, based on its unique four-wave longitudinal design, its sophisticated analytical approach based on parallel process modeling, and its explicit test of two relevant (*buffer* or *brake*) hypotheses.

## Conclusion

This study shows that parents play a role in how quickly adolescents transition toward increasingly permissive sexual attitudes and advanced sexual behavior and sexual risk behavior. Although our data seem to suggest that parent–child communication about sex is oftentimes “after the fact”, we also find that more directive parental communication that conveys love-and-respect oriented sexual norms can brake adolescents’ move toward sexual maturity. Contrary to a dominant hypothesis among scholars, and perhaps to popular belief as well, our stringent longitudinal analyses provided no evidence for a sexualized media buffering effect of parents’ instructive mediation. Instead, adolescents’ sexualized media consumption and sexual development appear to develop in tandem, as different components of one underlying transition toward sexual maturity. Our data suggest that this transition may already start in early adolescence, identifying early adolescence as a key developmental phase for implementing possible support programs aimed at helping parents to communicate about sex with their children timely and effectively.

## Disclaimer

All procedures performed in this study were in accordance with the ethical standards of the institutional review board of the Faculty of Social Sciences of Utrecht University and with the 1964 Helsinki declaration and its later amendments or comparable ethical standards. Furthermore, the present study was conducted in accordance with the ethical standards for studies involving human participants under Dutch law.
